# Redox Proteomic Profile of Tirapazamine-Resistant Murine Hepatoma Cells

**DOI:** 10.3390/ijms24076863

**Published:** 2023-04-06

**Authors:** Aušra Nemeikaitė-Čėnienė, Per Haberkant, Dalius Kučiauskas, Frank Stein, Narimantas Čėnas

**Affiliations:** 1 State Research Institute Center for Innovative Medicine, Santariškių St. 5, LT-08406 Vilnius, Lithuania; ausra.ceniene@imcentras.lt; 2 Proteomics Core Facility EMBL Heidelberg, Meyerhofstrasse 1, 69117 Heidelberg, Germany; per.haberkant@embl.de (P.H.); frank.stein@embl.de (F.S.); 3 Department of Xenobiotics Biochemistry, Institute of Biochemistry of Vilnius University, Saulėtekio 7, LT-10257 Vilnius, Lithuania; dalius.kuciauskas@gmail.com

**Keywords:** tirapazamine, reductive activation, oxidative stress, cytotoxicity, cell resistance

## Abstract

3-Amino-1,2,4-benzotriazine-1,4-dioxide (tirapazamine, TPZ) and other heteroaromatic *N*-oxides (ArN→O) exhibit tumoricidal, antibacterial, and antiprotozoal activities. Their action is attributed to the enzymatic single-electron reduction to free radicals that initiate the prooxidant processes. In order to clarify the mechanisms of aerobic mammalian cytotoxicity of ArN→O, we derived a TPZ-resistant subline of murine hepatoma MH22a cells (resistance index, 5.64). The quantitative proteomic of wild-type and TPZ-resistant cells revealed 5818 proteins, of which 237 were up- and 184 down-regulated. The expression of the antioxidant enzymes aldehyde- and alcohol dehydrogenases, carbonyl reductases, catalase, and glutathione reductase was increased 1.6–5.2 times, whereas the changes in the expression of glutathione peroxidase, superoxide dismutase, thioredoxin reductase, and peroxiredoxins were less pronounced. The expression of xenobiotics conjugating glutathione-S-transferases was increased by 1.6–2.6 times. On the other hand, the expression of NADPH:cytochrome P450 reductase was responsible for the single-electron reduction in TPZ and for the 2.1-fold decrease. These data support the fact that the main mechanism of action of TPZ under aerobic conditions is oxidative stress. The unchanged expression of intranuclear antioxidant proteins peroxiredoxin, glutaredoxin, and glutathione peroxidase, and a modest increase in the expression of DNA damage repair proteins, tend to support non-site-specific but not intranuclear oxidative stress as a main factor of TPZ aerobic cytotoxicity.

## 1. Introduction

**Scheme 1:**
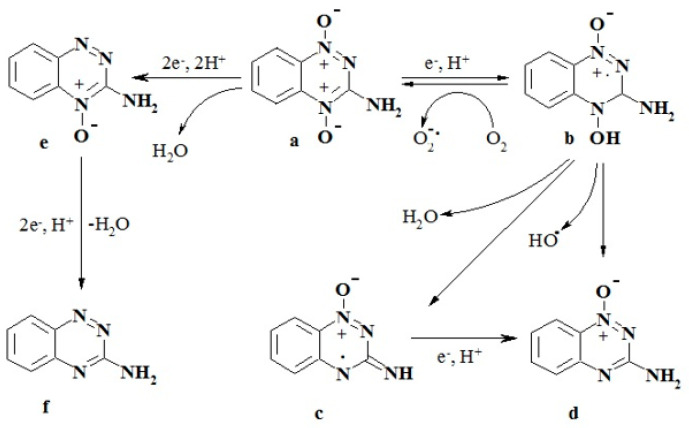
Pathways of the reductive transformation of tirapazamine (**a**) in the cell. Reduction products: TPZ anionradical (**b**), TPZ benzotriazinyl radical (**c**), TPZ 1-*N*-oxide (**d**), TPZ 4-*N*-oxide (**e**), and TPZ nor-oxide (**f**).

The derivatives of 3-amino-1,2,4-benzotriazine-1,4-dioxide (tirapazamine, TPZ) and other heteroaromatic *N*-oxides (ArN→O) exhibited antitumor, antibacterial, and antiprotozoal activities, and were considered potential hypoxia-selective antitumor agents [1,2,3,4,5,6,7]. Their action was typically attributed to enzymatic reductive activation and free radical generation. In the cell, TPZ (Scheme 1, structure a) was reduced in a single-electron way to a free radical (Scheme 1, structure b), which, under aerobic conditions, participates in redox cycling with the formation of O_2_**^−•^**, thereby causing oxidative stress-type cytotoxicity [1,6,7]. Under hypoxic conditions, free radical (b) forms a highly reactive benzotriazinyl radical (Scheme 1, structure c) that abstracts an H atom from DNA ([8,9], and references therein) and/or an oxidizing hydroxyl radical (OH^•^) ([6,10], and references therein). The nature of DNA-damaging species remains a matter of debate. The final relatively nontoxic products of the hypoxic metabolism of TPZ are its 1-*N*-oxide and nor-oxide (Scheme 1, structures d and f), which are possibly formed via free radical intermediates and 4-*N*-oxide intermediate (structure e), respectively [11].

The mechanisms of aerobic ArN→O cytotoxicity have been studied less well than hypoxic due to their less pronounced effect. However, it represents an important problem because some ArN→O exhibit anticancer activities under aerobic conditions at their micromolar concentrations [12,13]. A major contributor to ArN→O toxicity in aerobic mammalian cell cultures and oxygenated tissues is believed to be oxidative stress, which is induced by their redox cycling. This is indicated by non-respiratory O_2_ consumption and H_2_O_2_ production by the cells [14], lipid peroxidation [15], and the protective effects of antioxidants and Fe ion chelators [7]. Redox cycling may also be responsible for the antiparasitic and antibacterial effects of ArN→O [4,16,17] and may accompany their use in veterinary medicine. In terms of mechanisms, it is almost universally accepted that the most important cellular flavoenzyme performing a single-electron reduction in ArN→O and responsible for both their aerobic and hypoxic cytotoxicity is NADPH: cytochrome P450 reductase (POR) [18,19]. The role of other reductases is debatable (insufficiently characterized nuclear NAD(P)H-dehydrogenases [20,21,22]) and is of moderate importance with (NAD(P)H:quinone oxidoreductase (NQO1), catalyzing the mixed single- and two-electron reduction in ArNO [23,24]), or poorly studied (cytochromes P450 [25,26]). The study of TPZ-insensitive A549 cells also partly revealed the role of antioxidant enzymes since, in addition to decreased POR and NQO1 activity, superoxide dismutase (SOD) and glutathione reductase (GR) activity was increased, although catalase (CAT) activity increased modestly [27]. The overexpression of POR or deletion of SOD in cells sensitizes them to the effects of TPZ [28]. However, the obtained information concerned only limited groups of redox enzymes.

More recently, the genomic and proteomic studies of *Saccharomyces cerevisiae* [29], *Escherichia coli* [16], human adrenocortical carcinoma NCI-H295R cells [30], and *Entamoeba histolytica* [17] identified a number of DNA-repair, transport and redox proteins that were responsible for resistance to ArN→ O. Among the latter, the increased expression of SOD [16], thioredoxin and peroxiredoxin [17,30], and cytochrome P450 21A2 [30] could be mentioned. Continuing in this direction and aiming to characterize the redox proteins that are responsible for the resistance of mammalian cells to TPZ, we derived a TPZ-resistant subline of murine hepatoma MH22a cells and performed its proteomic characterization. Among other data, we identified aldehyde dehydrogenases and carbonyl reductases as the groups of antioxidant enzymes conferring cell resistance to TPZ.

## 2. Results

### 2.1. Generation and Characterization of TPZ-Resistant MH22a Cell Subline

Our previous studies showed that the murine hepatoma MH22a cells are sufficiently sensitive to TPZ under *aerobic* conditions [7,24]. The TPZ-resistant MH22a cell subline was obtained as described in the Materials and Methods. We were unable to obtain a cell subline in a stepwise fashion by growing the cells in increasing concentrations of TPZ because its concentration increased by up to 10 μM and caused cell death after 1 month. The resulting cell subline was resistant to TPZ (cL_50_ = 175 ± 15 μM vs. cL_50_ = 31 ± 4.0 μM of the parental line) and H_2_O_2_ (cL_50_ = 170 ± 15 μM vs. cL_50_ = 76 ± 6.0 μM), but equally sensitive to daunorubicin (cL_50_ = 6.0 ± 0.5 μM). The resistant subline, characterized by a doubling time of 101 ± 15 h (15–20th passages, 5.0 µM TPZ), proliferated significantly slower than the parental line with a doubling time of 35 ± 2 h.

### 2.2. Identification of Proteins Differently Expressed in Parental and TPZ-Resistant MH22a Cells

**Figure 1:**
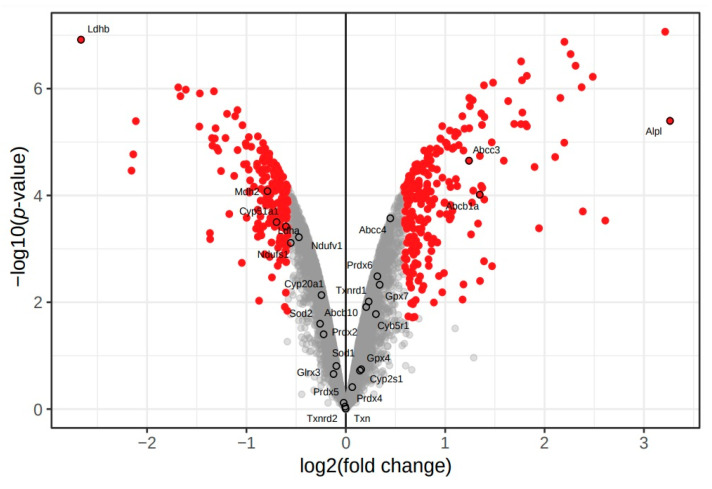
The volcano plot illustrates significantly changed protein levels in TPZ-resistant MH22a cells compared to the parental cell line. Highlighted in red are significantly down- and upregulated proteins (fold-change of at least 50% and false discovery rate < 0.05). Proteins not listed in Table 1, Table 2 and Table 3 but those mentioned in the text are marked with empty black circles.

In order to track down changes in the protein abundance of parental and TPZ-resistant MH22a cells (three biological replicates, *n* = 3), we performed a quantitative proteomic analysis using tandem mass tags (TMT). In total, we identified 7947 proteins, of which 5818 were identified with at least two unique peptides (Supplement S1). A total of 237 proteins were significantly increased in resistant cells (the change in protein level was greater than 1.5-fold, with a false discovery rate that was smaller than 0.05), and 184 were decreased in protein abundance (Figure 1). Among the enzymes with the most pronounced expression changes in TPZ-resistant cells, alkaline phosphatase (Alpl) increased 9.6-fold (Figure 1). It has also been shown that this enzyme was upregulated under conditions of oxidative stress [31]. It is possible that it is involved in the regulation of polyphosphate: the amount of which also increases under prooxidant conditions [32,33].

Among downregulated enzymes, the expression of lactate dehydrogenase B (Ldhb) decreased the most by 6.34 times (Figure 1, Table S1). This can be explained by the slowing down of glycolysis [34], which, in turn, increases cell doubling time. In addition, other glycolytic enzymes were also decreased: glucose-6-phosphate-dehydrogenase—2.54 times (G6px), malate dehydrogenase—1.72 times (Mdh2), lactate dehydrogenase A—1.52 times (Ldha) (Table S1).

Here, we aimed for a systematic mapping of expressional changes in enzymes related to a reduction in prooxidant xenobiotics, their detoxification, as well as protection against oxidative stress. For the general assessment of protein categories that are responsible for their resistance to TPZ, we performed gene ontology (GO) enrichment analysis using the clusterProfiler R package [35]. This tool applies biological term classifications and their enrichment, thus helping to better understand higher-order functions of the biological system. GO enrichment analysis performed for the cellular compartment (CC), molecular function (MF), and biological processes (BP) showed that cell resistance to TPZ was associated with increased glutathione transferase, aldehyde dehydrogenase, response to xenobiotics, and xenobiotic metabolizing activity (Figures S1–S3). Further, using DAVID, the protein functional clusters were obtained according to annotations GO:0016491 (oxidoreductase activity) (Table 1), GO:0006979 (response to oxidative stress) (Table 2), GO:0006805, GO:0024178, and GO:0006749 (xenobiotics metabolism and catabolism, and glutathione metabolism) (Table 3). It may be observed that the resulting clusters could partly overlap, evidently due to several functions of the enzymes. When possible, the protein expression changes were compared with the data of the proteomic analysis of the MH22a subline, which was insensitive to another redox active antitumor agent, 2,5-diaziridinyl-3-hydroxymethyl-6-methyl-1,4-benzoquinone (RH1) [36] (Table 1, Table 2 and Table 3).

**Table 1:** Differently expressed oxidoreductase proteins in TPZ-resistant MH22a cells.

No.	Gene	Uniprot Accession Code	Protein	Fold Change in TPZ-Resistant Cells	Fold Change in RH1-Resistance Cells [36]
1	2	3	4	5	6
Upregulated					
1	Aldh3a1	P47739	Aldehyde dehydrogenase, dimeric NADP-preferring	5.18	
2	Aldh1a1	P24549	Retinaldehyde dehydrogenase 1	4.97	6.00
3	Adh7	Q64437	Alcohol dehydrogenase class 4 mu/sigma chain	4.80	1.16
4	Aldh3b1	Q3TX25	Aldehyde dehydrogenase family 3 member B1	3.43	
5	Aldh1a7	B2RTL5	Aldehyde dehydrogenase family 1	3.42	5.16
6	Gsto1	O09131	Glutathione S-transferase omega-1	2.63	0.90
7	Cbr3	Q8K354	Carbonyl reductase (NADPH) 3	2.62	1.54
8	Adh1	P00329	Alcohol dehydrogenase 1	2.43	
9	Ugdh	O70475	UDP-glucose 6-dehydrogenase	2.26	
10	Cat	P24270	Catalase	2.00	0.59
11	Oxr1	E9Q0A7	Oxidation resistance protein 1	1.77	
12	Gsr	P47791	Glutathione reductase, mitochondrial	1.77	0.73
13	Aldh3a2	B1ATI0	Fatty aldehyde dehydrogenase	1.76	
14	Selenbp2	P17563	Selenium-binding protein 2	1.75	
15	Cryl1	A7VMV2	Crystallin, lambda 1, isoform CRA	1.73	
16	Dcxr	A2AC16	Dicarbonyl L-xylulose reductase, isoform CRA	1.72	
17	Blvrb	Q3U6G1	Biliverdin reductase B (Flavin reductase, NADPH)	1.67	0.40
18	Ptgr1	Q91YR9	Prostaglandin reductase 1	1.66	
19	Cyp4f16	Q99N17	Cytochrome P450 4F16	1.64	
20	Acox1	A2A848	Peroxisomal acyl-coenzyme A oxidase 1	1.60	
21	Cbr1	B2RXY7	Carbonyl reductase 1	1.59	1.32
22	Nqo1	Q542Y0	NAD(P)H dehydrogenase (quinone)	1.55	<0.10
23	Pnpo	Q91XF0	Pyridoxine-5’-phosphate oxidase	1.53	
Downregulated					
24	Nsdhl	Q3US15	NAD(P) dependent steroid dehydrogenase-like protein	0.66	
25	Ldha	P06151	L-lactate dehydrogenase A	0.66	1.27
26	Vkorc1	Q0VGU5	Vitamin K epoxide reductase complex subunit 1-like protein	0.66	
27	Bckdhb	Q6P3A8	2-oxoisovalerate dehydrogenase subunit beta, mitochondrial	0.65	
28	Pycrl	Q9DCC4	Pyrroline-5-carboxylate reductase 3	0.65	
29	Mthfd1	Q922D8	C-1-tetrahydrofolate synthase, cytoplasmic	0.65	
30	Cyp51a1	Q8K0C4	Lanosterol 14-alpha demethylase	0.62	
31	Mdh2	P08249	Malate dehydrogenase, mitochondrial	0.58	1.30
32	Fasn	P19096	Fatty acid synthase	0.57	
33	Dhcr7	O88455	7-dehydrocholesterol reductase	0.57	
34	Hsd17b12	O70503	Very-long-chain 3-oxoacyl-CoA reductase	0.57	
35	Steap1	Q9CWR7	Metalloreductase STEAP1	0.55	
36	Ndufb11	O09111	NADH dehydrogenase (ubiquinone) 1 beta-subcomplex subunit 11	0.54	
37	Por	P37040	NADPH-cytochrome P450 reductase	0.49	1.38
38	Dhfr	P00375	Dihydrofolate reductase	0.49	
39	Plod3	Q9R0E1	Procollagen-lysine,2-oxoglutarate 5-dioxygenase 3	0.47	
40	Cox4i2	Q91W29	Cytochrome c oxidase subunit 4 isoform 2,	0.47	
41	Degs1	O09005	Sphingolipid delta(4)-desaturase	0.44	
42	Sqle	P52019	Squalene monooxygenase	0.41	
43	G6px	Q00612	Glucose-6-phosphate 1-dehydrogenase X	0.39	0.85
44	Ptgs2	Q05769	Prostaglandin G/H synthase 2	0.23	
45	Ptgs1	P22437	Prostaglandin G/H synthase 1	0.22	
46	Ldhb	P16125	L-lactate dehydrogenase B chain	0.16	1.25

**Table 2:** Differently expressed oxidative stress response proteins in TPZ-resistant MH22a cells.

No.	Gene	Uniprot Accession Code	Protein	Fold Change in TPZ-Resistant Cells	Fold Change in RH1- Resistant Cells [36]
1	Aldh1a1	P24549	Retinaldehyde dehydrogenase 1	4.97	6.00
2	Aldh3b1	Q3TX25	Aldehyde dehydrogenase family 3 member B1	3.43	
3	Gclc	P97494	Glutamate-cysteine ligase catalytic subunit	3.40	
4	Apoe	P08226	Apolipoprotein E OS = Mus musculus	2.17	
5	Cat	P24270	Catalase	2.00	0.59
6	Usp25	P57080	Ubiquitin carboxyl-terminal hydrolase 25	1.88	
7	Oxr1	E9Q0A7	Oxidation resistance protein 1	1.77	
8	Nqo1	Q542Y0	NAD(P)H dehydrogenase [quinone]	1.55	<0.1

**Table 3:** Differently expressed enzymes of xenobiotics metabolism and catabolism, and glutathione metabolism in TPZ-resistant MH22a cells.

No.	Gene	Uniprot Accession Code	Protein	Fold Change in TPZ- Resistant Cells	Fold Change in RH1-Resistant Cells [36]
1	Gclc	P97494	Glutamate-cysteine ligase catalytic subunit	3.40	
2	Gsto1	O09131	Glutathione S-transferase omega-1	2.63	0.90
3	Gstm5	E9PVM7	Glutathione S-transferase Mu 5	2.57	
4	Gstm1	P10649	Glutathione S-transferase Mu	2.38	0.58
5	Ahr	P30561	Aryl hydrocarbon receptor	2.38	
6	Gsta4	P24472	Glutathione S-transferase A4	1.99	
7	Gsr	P47791	Glutathione reductase, mitochondrial	1.77	0.73
8	Cyp4f16	Q99N17	Cytochrome P450 4F16	1.64	
9	Gstm2	P15626	Glutathione S-transferase Mu 2	1.64	
10	Cbr1	B2RXY7	Carbonyl reductase 1	1.59	
11	Nceh1	Q8BLF1	Neutral cholesterol ester hydrolase 1	1.58	

Other enzymes potentially important in TPZ cytotoxicity can be mentioned in this context (Figure 1, Table S1). The changes in the expression of other flavoenzymes, which may potentially reduce TPZ, are less significant: NADH:cytochrome *b*_5_ reductase (Cyb5r1)—1.23, NADPH:adrenodoxin reductase (Fdxr)—1.32, and mitochondrial NADH: ubiquinone reductase (Ndufv1 and Ndufs1)—0.72 and 0.68 times, respectively. The protein levels of cytochromes P450, which could contribute to TPZ cytotoxicity [25,26], varied insignificantly, from 1.10 (Cyp2s1) to 0.84 (Cyp20a1) and 0.62 (Cyp51a1). The number of other antioxidant enzymes and proteins that could be upregulated during the response to oxidative stress changed insignificantly: glutathione peroxidase—1.27–1.11 (Gpx7, Gpx4), peroxiredoxin—1.25–0.86 (Prdx6, Prdx4, Prdx5, Prdx2), thioredoxin reductase—1.17 (Txnrd1), 1.0 (Txnrd2), thioredoxin (Txn)—1.0, superoxide dismutase—0.94 (Sod1), 0.84 (Sod2), glutaredoxin (Glrx3)—0.92. Among the other, potentially relevant groups of enzymes, the expression of a transcription factor aryl hydrocarbon receptor (Ahr) was increased by 2.38 times, and changes in the expression of multidrug resistance proteins and transporters were variable—2.54 (Abcb1a), 2.36 (Abcc3), 1.36 (Abcc4), and 1.15 (Abcb10).

### 2.3. Determination of Activities of Redox Enzymes in Parental and TPZ-Resistant MH22a Cells

For additional validation of protein expression changes, we determined the activities of redox enzymes that were potentially relevant to TPZ cytotoxicity in parental and TPZ-resistant MH22a cells (Table 4).

Table 4 shows that there exists a parallelism between the changes in the enzyme activity and the protein expression profiles (Table 1), namely, the almost unchanged activity of SOD, the elevated activities of CAT, GR, NQO1, and aldehyde dehydrogenases, and decreased activities of POR and lactate dehydrogenase.

## 3. Discussion

To the best of our knowledge, this was the first proteomic study of TPZ-insensitive mammalian cells, which complements the previously obtained data in the A549 TPZ-resistant cell subline [27]. Both sublines possess a decreased prooxidant character, i.e., they have a decreased content of POR (Table 1) [27], which is supposed to be the most important flavoenzyme responsible for a single-electron reduction in TPZ and its subsequent redox cycling [8,19] (Scheme 1). In the TPZ-insensitive MH22a subline, other enzymes capable of a one-electron reduction in TPZ, e.g., Steap1, Ndufb11 (Table 1), and Cyb5r1 and Fdxr were identified for the first time; however, their expression changed less significantly and in both directions. As in the TPZ-insensitive A549 subline, the MH22a subline has an increased content of the antioxidant enzyme GR (Table 1). However, there exist certain differences: the amount of SOD remained almost unchanged, while it increased several times in the A549 subline. By contrast, the amount of CAT in the resistant MH22a cells increased more (Table 1) than that of A549 cells. The overall increase in antioxidant activity was reflected in the 2.2-fold increased resistance of the TPZ-resistant subline to H_2_O_2_. Compared to other research subjects, proteomic data also showed that thioredoxins and peroxiredoxins were induced upon the exposure of *E. histolytica* and NCI-H295 cells to ArN→O [17,30], which is uncharacteristic of TPZ-insensitive MH22a cells. However, the former subjects were studied under different conditions, exposing them for a short time to relatively high concentrations of ArN→O, which, in the case of NCI-H295 cells, caused significant death [30].

However, our main finding is the enhanced expression of aldehyde dehydrogenases Aldh3a1, Aldh1a1, Aldh3b1, and Aldh1a7, and carbonyl reductases Cbr3 and Cbr1 observed in TPZ-resistant MH22a cells (Table 1), which represent an additional antioxidant enzyme defense mode. Aldehyde dehydrogenases and carbonyl reductases convert aldehydes derived from lipid peroxidation into the corresponding carboxylic acids and alcohols, respectively, thus contributing to their detoxification [37,38,39,40,41]. This phenomenon was partly evidenced in RH1-resistant MH22a cells (Table 1), where the redox cycling of RH1 partly contributed to its cytotoxicity [36]. However, it was not demonstrated in the previous studies of TPZ-resistant A549 subline [27] and microorganisms [16,17,29]. This finding may clarify some details on the roles of site-specific redox cycling in the aerobic cytotoxicity of TPZ (Scheme 1). At the time of writing, it is unclear whether the main factor in TPZ *hypoxic* cytotoxicity [18,19,20,21] is TPZ reduction by POR, localized in the cytoplasmic reticulum, or its reduction by uncharacterized intranuclear flavoenzymes. On the other hand, it was suggested that the *aerobic* cytotoxicity of TPZ was mainly governed by its single-electron reduction and redox cycling in the nucleus, i.e., close to the pool of DNA [22]. However, this suggestion was based on the targeted expression of POR in various cell compartments [22] and not on the functioning of enzymes under natural conditions. Our data show that in the resistant cells, the expression of nuclear antioxidant proteins Prdx2, Gpx4, and Glrx3 [42] was altered only slightly. On the other hand, the expression of aldehyde dehydrogenases, which were located mainly in the cytoplasm [37,39], was enhanced more significantly (Table 1). In our opinion, these findings tend to support the non-site-specific redox cycling of TPZ as a main determinant of its aerobic cytotoxicity and point to the less significant than expected role of its intranuclear redox cycling [22]. There is also evidence for the redox cycling of TPZ in isolated mitochondria [28].

Our work also provides some information about changes in signal transduction in TPZ-resistant MH22a cells. In parallel with the increase in the expression of aldehyde dehydrogenases, GST, and, to some extent, NQO1 (Table 1), the expression of Ahr is also increased (Supplement S1). This transcription factor activates the expression of the mentioned proteins and also cytochromes P450 [43]. Besides regulating xenobiotics metabolism, Ahr has many functions and is considered a signaling protein [44]. There is also evidence that the interaction between Ahr and the antioxidant response element (ARE) via the nuclear factor E2-related factor 2 (Nrf2) can change the expression of antioxidant and xenobiotic metabolizing enzymes, including GST and NQO1 [45]. Importantly, Nrf2/ARE were activated by oxidative stress [46] and responsible for the induction of CAT, SOD, and GR [47]. However, although TPZ-resistant cells had significantly increased levels of CAT and GR (Table 1), Nrf2 was not identified in the current proteomic analysis. Thus, according to the available data, among the possible signaling pathways, the relationship between Ahr and the expression of antioxidants and xenobiotics when metabolizing enzymes remains the best elucidated. In this context, the increase in NQO1 levels in TPZ-resistant cells (Table 1) is somehow unexpected since NQO1 promotes Ar*N*→O cytotoxicity in short-time 24–48 h experiments [24]. However, it can be assumed that the increase in GST and NQO1 levels (Table 1) is the result of TPZ-induced oxidative stress, which lasts for several months in our conditions, and the cytotoxicity-promoting effect of NQO1 was compensated by other factors. This can be compared with the data of the proteomic analysis of the MH22a subline, which is insensitive to the antitumor benzoquinone RH1 [36] (Table 1). The cytotoxicity of RH1 is primarily determined by an NQO1-catalyzed two-electron reduction in the DNA-alkylating diaziridinyl-hydroquinone, with the secondary role of single-electron reduction and oxidative stress [36,48]. Therefore, it is reasonable that the amount of NQO1 and GST was reduced in RH1-resistant cells (Table 1 and Table 3).

## 4. Materials and Methods

### 4.1. Cell Growth and Preparation for Mass Spectrometry

Murine hepatoma cells MH22a obtained from the Institute of Cytology of the Russian Academy of Sciences (St. Petersburg, Russia) were grown at 37 °C in a DMEM medium and supplemented with 10% fetal bovine serum and antibiotics in T25 flasks until they reached 70–80% confluence. The medium was then changed to an analogous medium with 5.0 μM TPZ, and, depending on cell viability, 50% of the medium with TPZ changed 1–2 times a week, repeating this procedure for 3.5 months to form a stable monolayer. The resulting monolayer was inoculated 1:2 and further grown by changing 50% of the culture medium twice a week until re-inoculation. Twenty passages were made over 2.5 months in total. All procedures were performed under aerobic conditions. For analysis and cytotoxicity experiments, cells between the 15th and 20th passages were used. For analysis, one flask per sample, 3 samples of the parental cell line, and 3 samples of the TPZ-resistant cell line were selected. Cells were rinsed three times with PBS, detached with TrypLE Express (ThermoFisher Scientific, Waltham, MA, USA), and cell concentration and viability were determined with a Trypan blue and Countess 2 (ThermoFisher Scientific, Waltham, MA, USA) automated cell counter. The cells were rinsed by centrifuging 500× *g* for 5 min and were suspended in PBS three times. Finally, the cells were flash-frozen in liquid nitrogen and transferred to a −86 °C freezer for storage. TPZ was a generous gift of Dr. Jonas Šarlauskas (Institute of Biochemistry, Vilnius), and daunorubicin and H_2_O_2_ were obtained from Merck KGaG (Darmstadt, Germany).

### 4.2. Protein Sample Preparation and Mass Spectrometry Analysis

The cells were lysed in an SDS lysis buffer at 95 °C for 5 min. After cooling down, benzonase (Merck, Rahway, NJ, USA) was added, and the solution was incubated for 60 min at 37 °C. The protein concentration was determined using the BCA assay. To achieve a quantitative assessment of protein abundance, an in-solution tryptic digest followed by TMT-labeling was performed in subsequent steps: (a) 10 µg of each sample were subjected to in-solution tryptic digest according to a modified version of the Single-Pot Solid-Phase-enhanced Sample Preparation (SP3) protocol [49,50]; (b) the lysates were added to Sera-Mag Beads (Merck, Rahway, NJ, USA) in 10 μL 15% formic acid and 30 μL of ethanol. The binding of proteins was achieved by shaking for 15 min at room temperature. SDS was removed by 4 subsequent washes with 200 μL 70% ethanol; (c) proteins were digested overnight at room temperature with 0.4 μg of sequencing grade modified trypsin in 40 μL HEPES/NaOH, pH 8.4 in the presence of 1.25 mM TCEP and 5 mM chloroacetamide; (d) the beads were separated, washed with 10 μL of an aqueous solution of 2% DMSO and the combined eluates were dried down; (e) peptides were reconstituted in 10 μL of H_2_O and reacted for 1 h at room temperature with 80 μg of the TMT6plex label reagent dissolved in 4 μL acetonitrile [51]. An excess TMT reagent was quenched by the addition of 4 μL of an aqueous 5% hydroxylamine solution; (f) peptides were reconstituted in 0.1% formic acid, mixed to achieve a 1:1 ratio across all TMT-channels and purified by a reverse phase clean-up step (OASIS HLB 96-well μElution Plate, Waters, Etten-Leur, The Netherlands); (g) peptides were subjected to an offline fractionation under high pH conditions [49]; (h) the resulting 12 fractions were analyzed by LC-MS/MS using a 90 min gradient on an Orbitrap Fusion Lumos mass spectrometer (ThermoFisher Scientific, Waltham, MA, USA). To this end, the peptides were separated using an Ultimate 3000 nano RSLC system (Dionex, ThermoFisher Scientific, Waltham, MA, USA) equipped with a trapping cartridge (Precolumn C18 PepMap100, 5 mm, 300 μm i.d., 5 μm, 100 Å) and an analytical column (Acclaim PepMap 100. 75 × 50 cm C18, 3 mm, 100 Å) connected to a nanospray-Flex ion source. Peptides were separated on the analytical column with a constant flow of 0.3 µL/min, applying a 90 min gradient of 2–28% of solvent B (0.1% formic acid in acetonitrile) in A (0.1% formic acid in water) followed by an increase of 40% B. The Orbitrap Fusion Lumos was operated in the positive ion mode with a spray voltage of 2.4 kV and a capillary temperature of 275 °C. Full scan MS spectra with a mass range of 375–1500 m/z were acquired in the profile mode using a resolution of 60,000 (maximum fill time of 50 ms; AGC Target was set to Standard) and an RF lens setting of 30%. Fragmentation was triggered for 3 s cycle time for peptide-like features with charge states of 2–7 on the MS scan (data-dependent acquisition). Precursors were isolated using the quadrupole with a window of 0.7 m/z and were fragmented with a normalized collision energy of 36%. Fragment mass spectra were acquired in profile mode with an orbitrap resolution of 15,000. The maximum fill time was set to 54 ms. The AGC target was set to 200%. The dynamic exclusion was set to 60 s.

### 4.3. Data Processing

The acquired data were analyzed using IsobarQuant [52] and Mascot V2.4 (Matrix Science) and using a reverse UniProt FASTA Mus musculus database (UP000000589, downloaded 15 May 2016 with 59,550 entries) including common contaminants. The following modifications were taken into account: Carbamidomethyl (C, fixed), TMT10plex (K, fixed), Acetyl (N-term, variable), Oxidation (M, variable), and TMT10plex (N-term, variable). The mass error tolerance for the full scan MS spectra was set to 10 ppm and for MS/MS spectra to 0.02 Da. A maximum of 2 missed tryptic cleavages were allowed. A minimum of 2 unique peptides with a peptide length of at least seven amino acids and a false discovery rate below 0.01 were required on the peptide and protein level [53].

The raw output files of IsobarQuant (protein.txt—files) were processed using the R programming language (R Foundation, Vienna, Austria, http://www.R-project.org, accessed on 3 May 2021). Only proteins that were quantified with at least two unique peptides were considered for the analysis. A total of 5818 proteins passed this quality control filter. Raw TMT reporter ion intensities (signal_sum columns) were first cleaned for batch effects using the removeBatchEffect function of the limma package [54] and were further normalized using vsn (variance stabilization normalization [55]). The proteins were then tested for their differential expression using the limma package. The replicate information was added as a factor in the design matrix and given as an argument to the ‘lmFit’ function of limma.

### 4.4. Bioinformatic Analysis

The database for Annotation, Visualization, and Integrated Discovery (DAVID, Bioinformatics Resources (December 2021 version, http://david.ncifcrf.gov/home.jsp, accessed on 6 September 2022)) was employed for the functional annotation analysis of the up-and down-regulated proteins compared to a background of all identified proteins. DAVID was used to functionally annotate the proteins that clustered together according to GO (gene ontology) annotations for biological processes and molecular functions. For quantitative bioinformatic analysis, the change in the protein level by more than 1.5-fold with a false discovery rate (fdr) value < 0.05 was considered significant. For GO enrichment analysis, the ‘compareCluster’ function of the R package ‘clusterProfiler’ was used [35] together with the mouse database (‘org.Mm.eg.db’ doi: 10.18129/B9.bioc.org.Mm.eg.db). Significant hits from the differential abundance analysis using limma were classified into up- and downregulated proteins and tested for GO enrichment.

### 4.5. Cytotoxicity Experiments

In the cytotoxicity experiments, 3.0 × 10^4^/mL cells were seeded in 5-mL flasks either in the presence or in the absence of compounds (TPZ, H_2_O_2_, or daunorubicin) and were grown on glass slides at 37 °C in a DMEM medium, supplemented with 10% fetal bovine serum and antibiotics for 24 h. The same cultivation procedure was applied to both parental and TPZ-resistant MH22a cells. The adherent cells were counted under a light microscope. Typically, they did not accumulate Trypan blue, and their viability was 98.5–99.3%.

### 4.6. Enzyme Activity Determination

For the enzymatic analysis, MH22a cells and their resistant subline were grown until confluence, detached by trypsinization, twice washed with PBS, and sonicated on ice in four cycles of 20 s. The homogenate was centrifuged at 14,000× *g* for 45 min, and the resulting supernatant with an added 1.0 mM PMSF was used for enzymatic analysis. The protein concentration was determined according to the method of Bradford. All the spectrophotometric measurements were performed using a Cary60 UV-Vis (Agilent Technologies, Santa Clara, CA, USA) spectrophotometer at 37 °C in 0.1 M K-phosphate (pH 7.0) containing 1.0 mM EDTA, as described previously [56]. The activity of CAT was determined following the decomposition of 10 mM H_2_O_2_ (Δε_240_ = 0.04 mM^−1^cm^−1^). The activity of SOD was determined from the inhibition of the reduction of 100 µM nitrotetrazolium blue by xanthine oxidase/xanthine system monitored at 560 nm. The activity of NAD(P)H:oxidases was determined according to the rate of oxidation of 100 µM NAD(P)H (Δε_340_ = 6.2 mM^−1^cm^−1^). The activity of POR was determined according to the rate of reduction of 50 µM cytochrome *c* (Δε_550_ = 20 mM^−1^cm^−1^) in the presence of 100 µM NADPH. The activity of GR was determined according to the rate of the oxidation of 100 µM NADPH in the presence of 1.0 mM glutathione. The activity of NQO1 was determined following the rate of reduction for 50 µM cytochrome *c* in the presence of 10 µM 2-methyl-1,4-naphthoquinone and 100 µM NADPH, as a difference between the reduction rate in the absence of dicumarol and its presence (20 µM). In this assay, 0.01% Tween 20 and 0.25 mg/mL bovine serum albumin was used as NQO1 activators. The activity of other enzymes was determined in 0.1 M K-phosphate (pH 7.5) according to the rate of oxidation of 200 µM NADH by 2.0 mM pyruvate (lactate dehydrogenase), or the rate of formation NAD(P)H from 1.0 mM NAD(P)^+^ in the presence of 2.5 mM benzaldehyde (NADP^+^, aldehyde dehydrogenase 3A1) or 1.0 mM propionaldehyde (NAD^+^, aldehyde dehydrogenase 1A1) [38]. In these cases, the reaction rates were corrected for NAD(P)H:oxidase activities.

## 5. Conclusions

The first proteomic study of the TPZ-resistant mammalian cell subline reveals its increased antioxidant character, which was demonstrated by an increase in the activity of catalase, glutathione reductase, and aldehyde dehydrogenases. The upregulation of aldehyde dehydrogenases which were located mainly in the cytoplasm tended to support the non-site-specific redox cycling of TPZ as a main determinant of its aerobic cytotoxicity. Most possibly, the increased expression of antioxidant and xenobiotics metabolizing enzymes is associated with the action of an aryl hydrocarbon receptor. In parallel, there was observed the downregulation of NADPH:cytochrome P450 reductase, which presumably played a leading role in the reductive activation of TPZ.

## Figures and Tables

**Table 4:** The activities of redox enzymes in MH22a cells and their TPZ-resistant subline.

No.	Enzyme	Activity	
		Parental MH22a	TPZ-Resistant MH22a
1	Superoxide dismutase (SOD) ^a^	3.6 ± 0.3	3.3 ± 0.3
2	Catalase (CAT) ^b^ ***	111 ± 5.2	209 ± 6.4
3	Glutathione reductase (GR) ^c^ ***	86 ± 4.0	137 ± 5.8
4	NADH:oxidase ^c^ *	2.8 ± 0.2	2.1 ± 0.1
5	NADPH:oxidase ^c^	2.1 ± 0.1	1.9 ± 0.1
6	NADPH:cytochrome *c* reductase (POR) ^c^ ***	10.5 ± 0.6	5.0 ± 0.3
7	NAD(P)H:quinone oxidoreductase (NQO1) ^c^ **	140 ± 7.0	191 ± 9.0
8	Lactate dehydrogenase ^c^ ***	2910 ± 210	1290 ± 78
9	Aldehyde dehydrogenase 3A1 ^c^ ***	14.0 ± 1.3	66 ± 7.5
10	Aldehyde dehydrogenase 1A1 ^c^ ***	6.5 ± 0.5	37 ± 3.2

^a^ Unit/mg protein; one unit of enzyme activity is defined as the amount of protein needed to inhibit a reduction in nitroblue tetrazolium by 50%, ^b^ µmol × mg protein ^−1^ × min^−1^, ^c^ nmol × mg protein ^−1^ × min^−1^, * *p* ≤ 0.03, ** *p* ≤ 0.01, *** *p* ≤ 0.002.

## Data Availability

The mass spectrometry proteomics data have been deposited to the ProteomeXchange Consortium via the PRIDE [1] partner repository with the dataset identifier PXD039876.

## Supplementary Materials

The following supporting information can be downloaded at: https://www.mdpi.com/article/10.3390/ijms24076863/s1.
