# Supplementary material for: Redox Proteomic Profile of Tirapazamine-Resistant Murine Hepatoma Cells
Source: Int J Mol Sci. 2023 Apr 6;24(7):6863. doi: 10.3390/ijms24076863 (PMC10094930; doi:10.3390/ijms24076863)
Supplement: Supplementary file 1 [file ijms-24-06863-s001.zip › ijms-2023-suppl S2.pdf]

Data of Gene Ontology (GO) enrichment of TPZ-resistant MH22a cells

For GO enrichment analysis, the 'compareCluster' function of the R package 'clusterProfiler' was used [1] together with the mouse data base ('org.Mm.eg.db' doi: 10.18129/B9.bioc.org. Mm.eg.db). Significant hits from the differential abundance analysis using limma have been classified into up- and downregulated proteins and tested for GO enrichment. The odds ratio was calculated as the ratio between the 'GeneRatio' and the 'BgRatio' from the compareCluster results tables. Dot plots indicate −log10 of adjusted *p*-values as colour, and odds ratio as the circle size of dots. Only top 10 significantly regulated GO terms are shown (adjusted $p \leq 0.05$). The statistical comparison (R-P) distinguished between upregulated and downregulated hits, is given on the x-axis of the dot plots (Figures S1–S3).

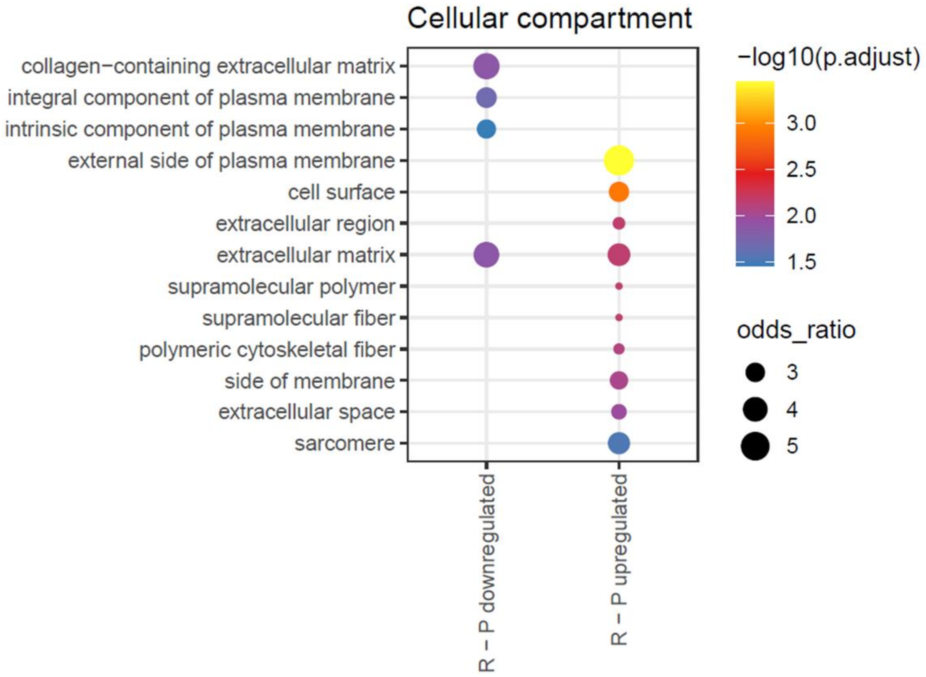

Figure S1. Protein expression changes in TPZ-resistant MH22a cells according to GO enrichment analysis, category cellular compartment.

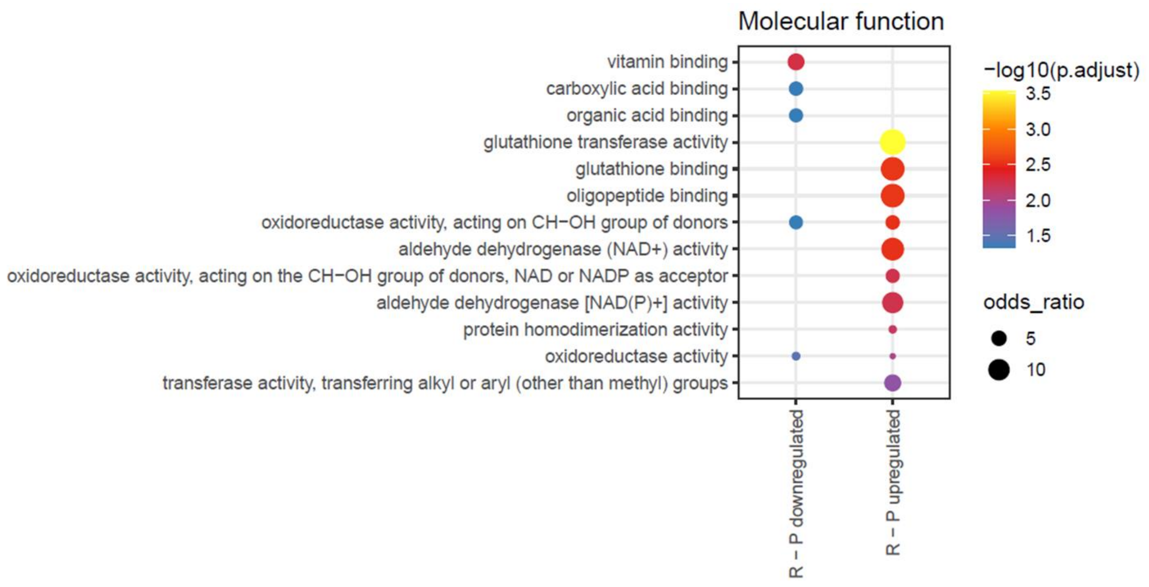

Figure S2. Protein expression changes in TPZ-resistant MH22a cells according to GO enrichment analysis, category molecular function.

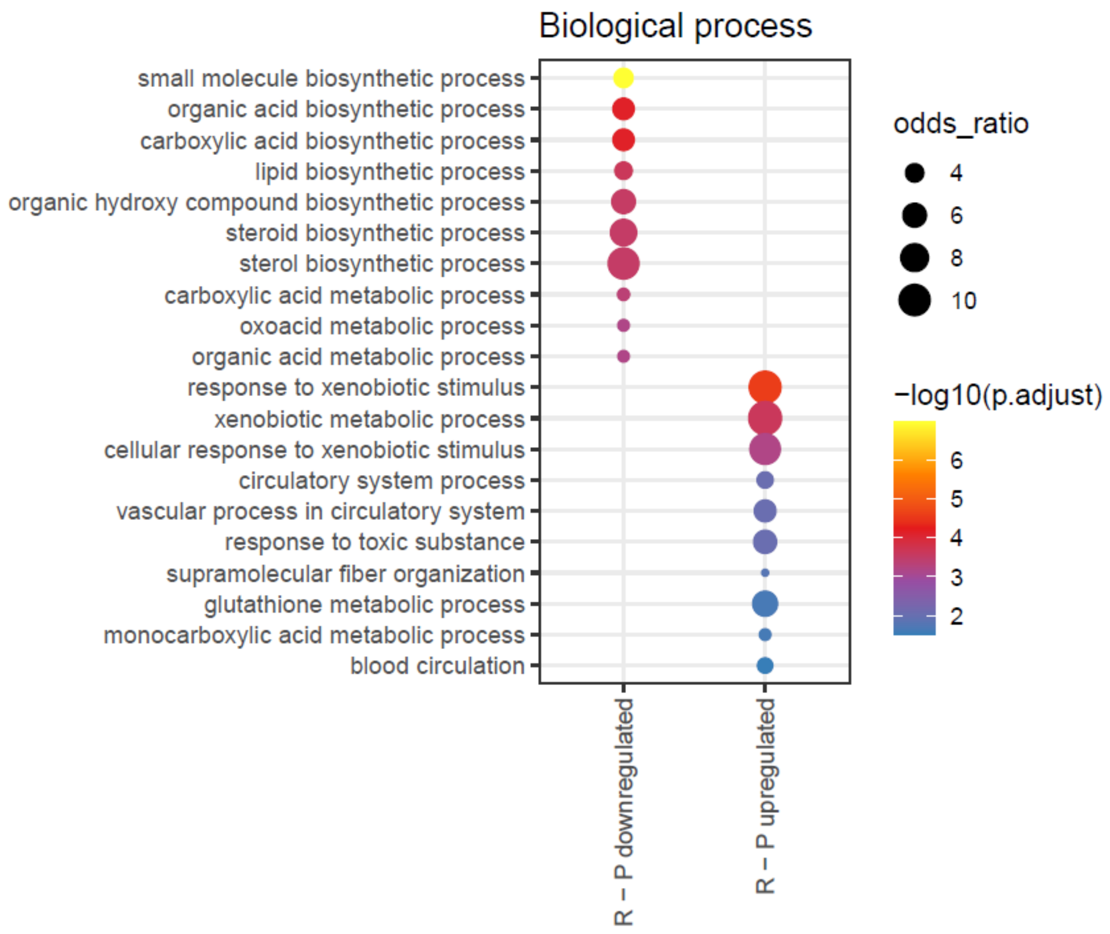

Figure S3. Protein expression changes in TPZ-resistant MH22a cells according to GO enrichment analysis, category biological process.

Reference

1. Yu, G.; Wang, L-G.; Han, Y.; He, Q-Y. clusterProfiler: an R package for comparing biological themes among gene clusters. *OMICS* **2012,** *16*, 284-287.
